# Liberal use of platelet transfusions in the acute phase of trauma resuscitation: a systematic review

**DOI:** 10.1186/cc11056

**Published:** 2012-03-20

**Authors:** J Hallet, F Lauzier, O Mailloux, V Trottier, P Archambault, R Zarychanski, AF Turgeon

**Affiliations:** 1 CHA-Hôpital de l'Enfant-Jésus, Université Laval, Québec, Canada; 2 Université Laval, Québec, Canada; 3 University of Manitoba, Winnipeg, Canada

## Introduction

With the recognition of early trauma coagulopathy, trauma resuscitation has recently shifted towards early and aggressive transfusion of platelets (PLTs). However, the clinical benefits of this strategy remain controversial. This systematic review examined the impact of an aggressive approach (higher PLT:RBC ratios) compared to restrictive PLT transfusions (lower PLT:RBC ratios) in the acute phase of trauma resuscitation.

## Methods

We systematically searched Medline, Embase, Web of Science, Biosis, Cochrane Central and Scopus to identify relevant randomized controlled trials (RCTs) and observational studies comparing the effect of two or more different PLT:RBC ratios in trauma resuscitation. We excluded studies using whole blood or systematically addressing the use of hemostatic products. Two independent reviewers selected the studies, extracted data using a standardized form, and assessed the risk of bias using the Newcastle-Ottawa scale and a checklist of key methodological elements (for example, use of massive transfusion protocol, survival bias). Disagreements were solved by consensus or a third party. The primary outcome was mortality. Secondary outcomes were multiple organ failure (MOF), lung injury and sepsis. A meta-analysis using random effects models was planned.

## Results

From 6,123 citations, seven observational studies were included (*n *= 4,230 patients). No RCT was identified. All studies were considered to be at low risk of bias and addressed confoundings through multivariate regression or propensity scores. Four studies (*n *= 1,978) reported a decrease in mortality with higher PLT:RBC ratios in patients requiring massive transfusion and one study observed no mortality difference (*n *= 1,181) in nonmassively transfused patients. Two studies reported on the implementation of a massive transfusion protocol with higher PLT:RBC ratios; only one revealed a survival benefit (*n *= 211). Of the three studies accounting for survival bias, two demonstrated a survival benefit (*n *= 1,300). Among two studies reporting on the secondary outcomes (*n *= 854), one observed an increase in MOF with higher PLT:RBC ratios. Clinical heterogeneity between studies and methodological limitations precluded the use of a meta-analysis.

## Conclusion

There is insufficient evidence to strongly support the use of a specific PLT:RBC ratio for acute trauma resuscitation, especially considering survival bias and nonmassively transfused patients. RCTs examining both safety and efficacy of liberal PLT transfusions are warranted.

